# Collargolised Catgut

**Published:** 1907-03

**Authors:** 


### Collargolised Catgut.

In the *Annals of Surgery,* Jan., 1907, Dr. J. E. Blake reports laboratory experimentation with collargised catgut, which shows that it has a marked inhibitory influence on bacterial growth.

The catgut is prepared as follows: four coils of catgut, each containing ten strands, are wound on four glass slabs and placed for a week in a jar containing a 2% collargolum solution. The jars are shaken once or twice in the intervals. The slabs are then taken out, washed in sterile water till the excess of collargol is removed, and placed in 95% alcohol for 15 to 30 minutes. Then the individual strands are wound on separate spools, under aseptic precautions, and preserved in 95% alcohol till used.

Dr. Pilcher adds: silverised catgut has since been used in all my operations (more than 500) at the Seney Methodist Episcopal Hospital. It has continued to give me abundant satisfaction and to justify all expectations. There has been a notable absence of infective accidents in an active general service which includes nearly every variety of operative interference. Silverised catgut has become established as a permanent factor in our operating room methods.

